# PARP1 regulates the protein stability and proapoptotic function of HIPK2

**DOI:** 10.1038/cddis.2016.345

**Published:** 2016-10-27

**Authors:** Jong-Ryoul Choi, Ki Soon Shin, Cheol Yong Choi, Shin Jung Kang

**Affiliations:** 1 Department of Molecular Biology, Sejong University, Seoul 143-747, Korea; 2 Department of Biology, Kyung Hee University, Seoul 130-701, Korea; 3 Department of Life and Nanopharmaceutical Sciences, Kyung Hee University, Seoul 130-701, Republic of Korea; 4 Department of Biological Sciences, Sungkyunkwan University, Suwon 440-746, Korea; 5 Department of Integrative Bioscience and Biotechnology, Sejong University, Seoul 143-747, Korea

## Abstract

Homeodomain-interacting protein kinase 2 (HIPK2) is a nuclear serine/threonine kinase that functions in DNA damage response and development. In the present study, we propose that the protein stability and proapoptotic function of HIPK2 are regulated by poly(ADP-ribose) polymerase 1 (PARP1). We present evidence indicating that PARP1 promotes the proteasomal degradation of HIPK2. The tryptophan-glycine-arginine (WGR) domain of PARP1 was necessary and sufficient for the promotion of HIPK2 degradation independently of the PARP1 enzymatic activity. The WGR domain mediated the interaction between HIPK2 and C-terminus of HSP70-interacting protein (CHIP) via HSP70. We found that CHIP can function as a ubiquitin ligase for HIPK2. The interaction between PAPR1 and HIPK2 was weakened following DNA damage. Importantly, PARP1 reduced the HIPK2-mediated p53 phosphorylation, proapoptotic transcriptional activity and cell death. These results suggest that PARP1 can modulate the tumor-suppressing function of HIPK2 by regulating the protein stability of HIPK2.

The homeodomain-interacting protein kinase 2 (HIPK2) is a serine/threonine kinase that has a critical role in the regulation of DNA damage response, cytokinesis, cell migration, differentiation and morphogenesis.^1, 2^ Under normal or hypoxic condition, the protein level of HIPK2 is maintained low by constant proteasomal degradation by E3 ubiquitin ligases such as seven in absentia homolog (SIAH1), SIAH2 and WD40 repeat/SOCS box-containing protein 1 (WSB1).^3, 4, 5^ However, HIPK2 can be stabilized by escaping from the proteasomal degradation under stress conditions such as DNA damage.^6^ It is known that DNA damage checkpoint kinases ataxia telangiectasia mutated (ATM), and ATM and Rad3-related (ATR) phosphorylate SIAH1, and thus disrupt the interaction between HIPK2 and SIAH1.^4, 5^ As the accumulation of HIPK2 can induce apoptosis, the regulation of HIPK2 protein stability is very important in determining the cell fate between survival and death.

HIPK2 is known as a tumor suppressor and has been reported to be inactivated in tumor cells.^7, 8^ HIPK2 induces apoptosis via p53-dependent or -independent manner. In the absence of p53, HIPK2 can mediate apoptosis following DNA damage via anti-apoptotic corepressor C-terminal binding protein (CtBP). When p53 is present, lethal DNA damage induces accumulation of HIPK2 and subsequent phosphorylation of p53 at serine 46 by HIPK2, which in turn activates apoptotic p53-target genes such as *PUMA*, *Bax* and *Noxa*.^9, 10^ Therefore, HIPK2 has been recognized as a target for cancer therapy.

Poly(ADP-ribose) polymerase 1 (PARP1) is a multifunctional nuclear enzyme that affects various aspects of cellular homeostasis including DNA repair, inflammation, cell proliferation and cell death.^11^ A major role of PARP1 is DNA repair and has been known as a ‘genomic guardian'.^12^ Supporting this view, inactivation or deletion of PARP1 induces a substantial level of genomic instability.^13^ In addition, previous reports showed that PARP1 promotes tumor cell survival by coactivating hypoxia-inducible factor 1*α* (HIF1*α*)-dependent gene expression.^14, 15^ Therefore, inhibitors of PARP1 have been tested as anticancer agents.

HIPK2 and PARP1 share functional working ground under DNA damage and interacting proteins such as p53, Groucho, HIF1 and p300.^9, 15, 16, 17, 18, 19, 20, 21^ However, it has not been studied whether there is an interplay between HIPK2 and PARP1. In the present study, we have investigated functional interaction of HIPK2 and PARP1. We found that PARP1 interacted with HIPK2 and promoted the HIPK2 polyubiquitination and proteasomal degradation, thus regulating the proapoptotic function of HIPK2. Our findings suggest that PARP1 has a critical role in the regulation of HIPK2 stability and thus cellular decision-making toward repair versus apoptosis.

## Results

### PARP1 colocalized and interacted with HIPK2

**Figure 1:**
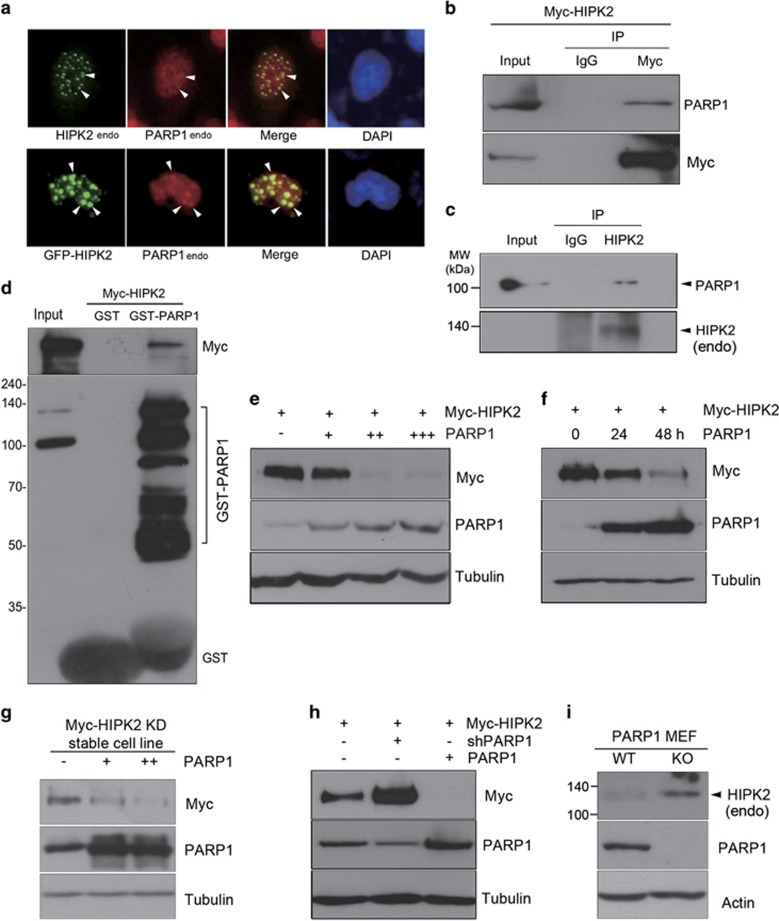
PARP1 interacted with HIPK2 and decreased its expression. (**a**) Representative images of HEK 293 cells stained for endogenous HIPK2 (green) and PARP1 (red) by indirect immunofluorescence (endo, endogenous). Overlapping localization is shown in yellow (merge). Nucleus was visualized by DAPI (upper panels). HEK 293 cells were transfected with expression vectors encoding GFP-HIPK2 and analyzed for HIPK2 (green) and endogenous PARP1 (red) by indirect immunofluorescence (lower panels). (**b**) Expression plasmid coding for Myc-HIPK2 was transfected into HEK 293 cells. The transfected cells were immunoprecipitated with anti-Myc antibodies, followed by immunoblotting using anti-PARP1 antibodies (upper panel). The blots were also examined for the detection of HIPK2 using anti-Myc antibodies (lower panel). The input lane represents 10% of total cell lysates. (**c**) Endogenous HIPK2 was immunoprecipitated from HEK 293 cell lysates using rabbit polyclonal anti-HIPK2 antibodies (endo) or control normal rabbit serum. The precipitates were analyzed by immunoblotting for the detection of PARP1 (upper panel) or HIPK2 (lower panel). (**d**) *In vitro* translated HIPK2 was incubated with equal amounts of either GST protein or purified GST-PARP1. Bound proteins were examined by immunoblotting using anti-Myc antibodies (upper panel). Affinity-purified GST or GST-PARP1 used in this assay is shown in the lower panel following blotting using anti-PARP1 and anti-GST antibodies. Input indicates 10% of *in vitro* translated Myc-HIPK2 used in the binding reaction. (**e**) Increasing amounts (0.125, 0.25, 0.5 *μ*g) of PARP1 expression vectors were transfected into HEK 293 cells with Myc-HIPK2 plasmids (0.5 *μ*g) and then examined by immunoblotting. (**f**) HEK 293 cells co-transfected with expression vectors for HIPK2 and PARP1 were incubated for the indicated times and then examined by immunoblotting. (**g**) PARP1 expression vector was transfected into the cell line stably expressing low level of kinase-dead Myc-HIPK2 (KD, K221R) and then analyzed by immunoblotting. (**h**) Myc-HIPK2 expression vector was transfected into HEK 293 cells with expression vector or shRNA vector (pLKO.1) for PARP1 to examine the effect of PARP1 knockdown on HIPK2 expression. (**i**) Wild-type (WT) and PARP1 knockout (KO) MEFs were prepared from three different mice per each genotype and pooled for culture. The levels of endogenous HIPK2 were analyzed by immunoblotting using anti-HIPK2 antibodies

To study whether there is a functional interplay between PARP1 and HIPK2, we first examined whether they colocalize in cells. As shown in Figure 1a, endogenous HIPK2 was colocalized with endogenous PARP1 in the nuclear speckle-like structures. Overexpressed HIPK2 was also colocalized with endogenous PARP1 (Figure 1a). In addition, both endogenous and overexpressed PARP1 and HIPK2 were co-immunoprecipitated (Figure 1b and c). To determine whether the interaction between HIPK2 and PARP1 is direct, we examined the interaction by GST pull-down assay. As shown in Figure 1d, GST-PARP1 was able to interact with the *in vitro* translated Myc-HIPK2, suggesting a direct interaction.

### PARP1 decreased the expression level of HIPK2

HIPK2 is unstable in unstressed cells but stabilized and activated following DNA damage to induce apoptosis.^9, 22, 23^ Meanwhile, PARP1 keeps the integrity of the genome and thus renders cells viable under normal conditions.^24^ Therefore, we hypothesized that there may be an antagonizing interplay between the two nuclear enzymes. First, we examined whether PARP1 affects the HIPK2 expression. As shown in Figure 1e and f, overexpression of PARP1 reduced the expression level of HIPK2. We then examined the effect of PARP1 overexpression on the protein level of HIPK2 in a cell line stably expressing low level of Myc-HIPK2 kinase-dead mutant. HIPK2 expression was decreased by PARP1 overexpression in the stable cell line as well (Figure 1g). To confirm whether endogenous PARP1 regulates HIPK2 expression, we examined the effect of PARP1 knockdown on the HIPK2 protein amount. In accordance with the overexpression data, knockdown of endogenous PARP1 markedly increased the HIPK2 expression (Figure 1h). In addition, the protein amount of endogenous HIPK2 was increased in the PARP1 knockout mouse embryonic fibroblasts (Figure 1i). Taken together, these results indicate that PARP1 decreases the expression level of HIPK2.

### PARP1 regulated the protein stability of HIPK2

**Figure 2:**
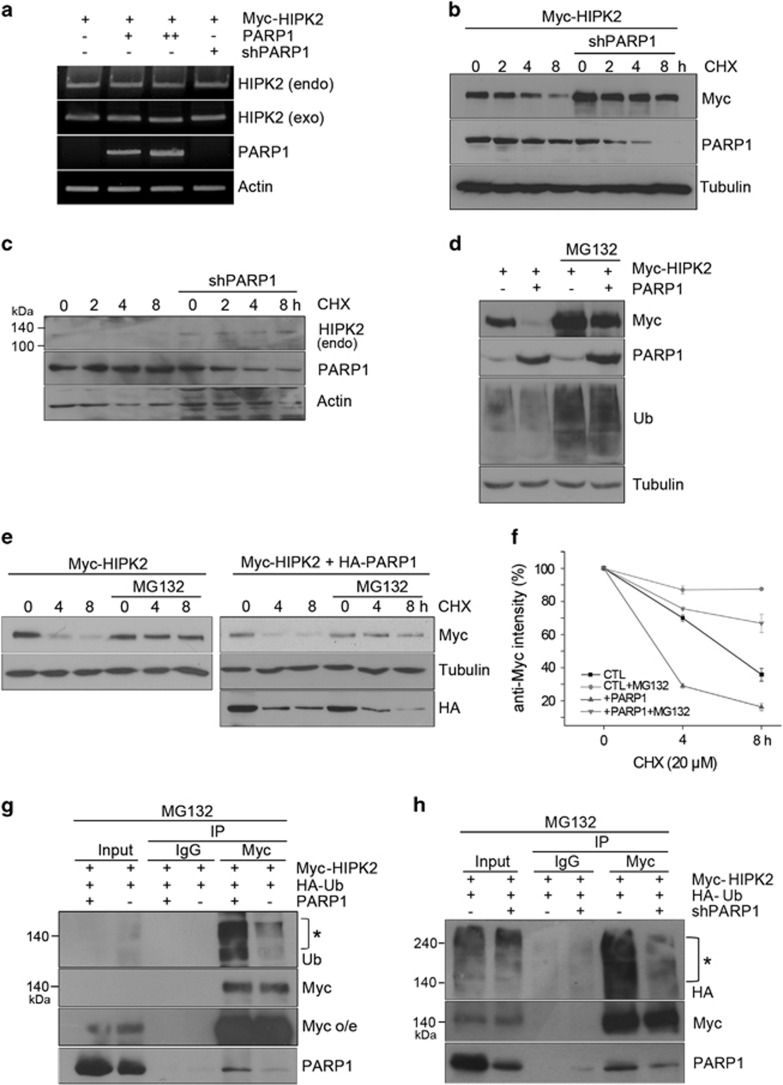
PARP1 regulated the protein stability of HIPK2. (**a**) The mRNA levels of both endogenous (endo, human) and exogenous (exo, mouse) HIPK2 were monitored by RT-PCR analysis following co-transfection of vectors for PARP1 (0.25 and 0.5 *μ*g) or pLKO.1 PARP1 shRNA (shPARP1). (**b** and **c**) The protein levels of overexpressed and endogenous HIPK2 were examined following PARP1 knockdown in the presence of cycloheximide (20 *μ*M). (**d**) HEK 293 cells were transfected with Myc-HIPK2 and empty vector or PARP1 expression vectors and then treated with 5 *μ*M of MG132 for 10 h. The cells were then processed for immunoblotting as indicated. (**e**) Expression vectors for Myc-HIPK2 with or without HA-PARP1 were expressed in HEK 293 cells and then the transfected cells were treated with 5 *μ*M of MG132. After 10 h, cells were further incubated with cycloheximide (20 *μ*M) and collected at the indicated time points. The cells were analyzed by immunoblotting using anti-Myc and anti-HA antibodies. (**f**) The Myc bands in E were quantified by densitometry. Control (CTL) represents Myc-HIPK2 overexpression. Means±S.D. of three independent experiments are shown. (**g** and **h**) To examine the changes in the ubiquitination of HIPK2 after altering PARP1 expression, Myc-HIPK2 and HA-Ubiquitin (HA-Ub) expression vectors were transfected into HEK 293 cells with PARP1 expression vector or shPARP1. At 24 h after the transfection, the cells were treated with MG132 (5 *μ*M) for 10 h. Next, the cell lysates were immunoprecipitated with anti-Myc antibodies, followed by immunoblotting using anti-Ub, anti-HA, anti-Myc and anti-PARP1. The input lane represents 10% of total cell lysates. Asterisks indicate the ubiquitinated Myc-HIPK2

To investigate at which level PARP1 regulates HIPK2 expression, we first examined whether PARP1 regulates the mRNA level of HIPK2. As shown in Figure 2a, PARP1 overexpression or knockdown did not affect the amount of HIPK2 mRNA, indicating that PARP1 posttranscriptionally regulates HIPK2 expression. It is well known that HIPK2 is an unstable protein with a high turnover rate in unstressed cells.^25^ Thus, we wanted to examine whether PARP1 regulates the protein stability of HIPK2. Protein stability of HIPK2 was monitored with or without PARP1 knockdown following inhibition of protein synthesis using cycloheximide. Both overexpressed and endogenous HIPK2 protein stability was increased when PARP1 was knocked down (Figure 2b and c). These results indicate that PARP1 regulates the protein stability of HIPK2.

As HIPK2 is known to be constantly degraded by proteasomes,^4, 5^ we then examined whether PARP1 regulates HIPK2 protein stability via proteasome-mediated degradation. We found that MG132 effectively inhibited the PARP1-dependent HIPK2 decrease (Figure 2d). We also examined whether the HIPK2 protein turnover promoted by PARP1 overexpression is suppressed by proteasome inhibition. Overexpression of PARP1 significantly decreased the half-life of HIPK2, but this was suppressed by MG132 treatment (Figure 2e and f). These results clearly indicate that PARP1 promotes proteasomal degradation of HIPK2.

To further examine whether PARP1 regulates proteasomal degradation of HIPK2, we assessed the ubiquitination level of HIPK2 after overexpression or knockdown of PARP1. As shown in Figure 2g, HIPK2 polyubiquitination was increased when PARP1 was overexpressed. Furthermore, PARP1 knockdown resulted in a decrease in polyubiquitination of HIPK2 (Figure 2h). Taken together, these results suggest that PARP1 promotes the degradation of HIPK2 mediated by ubiquitin-proteasome system.

### PARP1-induced HIPK2 degradation was independent of PARP1 enzymatic activity but mediated by WGR domain of PARP1

**Figure 3:**
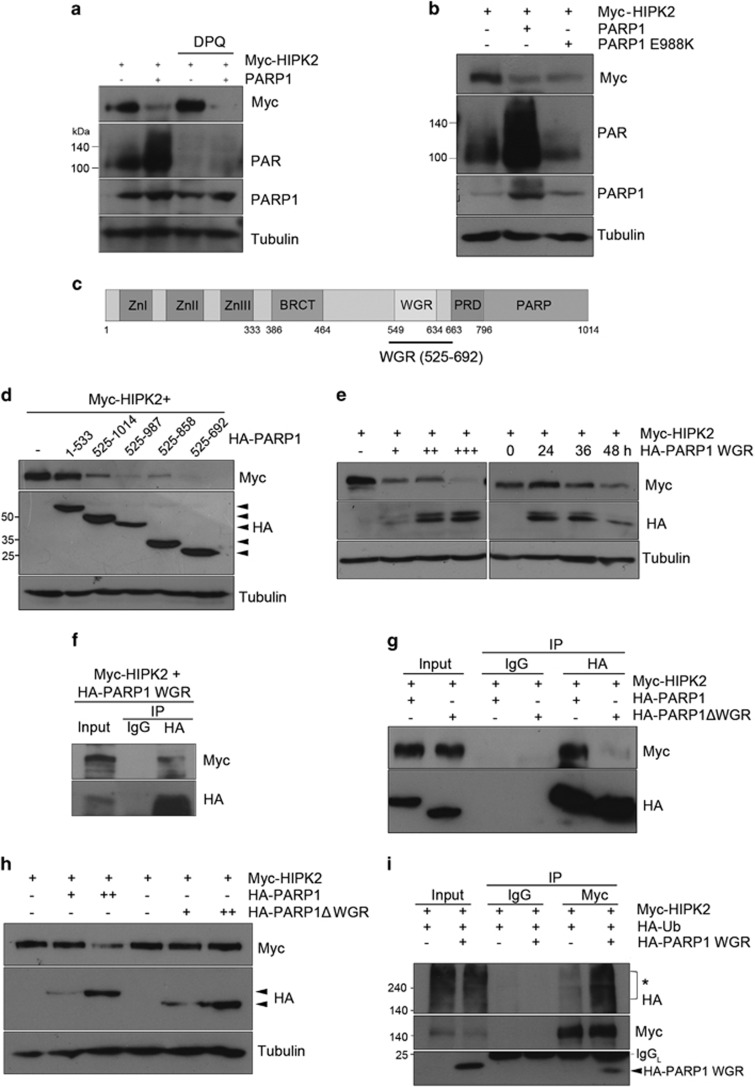
PARP1-induced HIPK2 degradation was independent of PARP1 activity but mediated by WGR domain of PARP1. (**a**) The cells transfected as indicated were treated with 50 *μ*M of DPQ and then examined by immunoblot. Blotting with anti-PAR was performed to confirm the activity of DPQ. (**b**) Myc-HIPK2 expression vector was transfected into HEK 293 cells along with either wild-type PARP1 or catalytically inactive mutant PARP1 (PARP1 E988K) and then examined by immunoblotting. (**c**) A schematic drawing of the PARP1 domains. Zn, zinc-finger domain; BRCT, breast cancer suppressor protein-1 domain; WGR, tryptophan-glycine-arginine domain, PRD, PARP-regulatory domain; PARP, PARP catalytic domain. (**d**) HEK 293 cells were transfected with Myc-HIPK2 expression vector and HA-tagged PARP1 wild-type or HA-PARP1 deletion mutants (amino acids 1–533, 525–1014, 525–987. 525–858 and 525–692) and then analyzed by immunoblotting. (**e**) HIPK2 expression vector was transfected into HEK 293 cells with increasing amounts of expression vector for HA-PARP1 WGR domain (amino acid 525–692; 0.125, 0.25, 0.5 *μ*g DNA, left panel) or with fixed amount of WGR expression vector (0.25 *μ*g DNA) for the indicated times (right panel). Protein expression of Myc-HIPK2 and HA-PARP1 WGR domain was analyzed by immunoblotting. (**f**) HEK 293 cells were co-transfected with HA-PARP1 WGR with Myc-HIPK2. Cell lysates were immunoprecipitated with anti-HA antibodies, followed by immunoblotting using anti-Myc and anti-HA antibodies. (**g**) HA-PARP1 or HA-PARP1ΔWGR (WGR deletion mutant) expression vector was transfected into HEK 293 cells in combination with Myc-HIPK2 expression vector. At 24 h after the transfection, the cells were treated with MG132 (5 *μ*M) for 10 h. After immunoprecipitation of PARP1 with anti-HA antibodies, co-precipitating Myc-HIPK2 protein was detected by immunoblotting (upper panel). The blot was then examined for the detection of PARP1 variants using anti-HA antibodies (lower panel). The input lane represents 10% of total cell lysates. (**h**) Myc-HIPK2 expression vector was transfected into HEK 293 cells along with increasing amounts (0.25 and 0.25 *μ*g DNA) of either HA-tagged WT PARP1 or PARP1 ΔWGR, followed by immunoblotting. (**i**) HEK 293 cells were transfected with expression vectors for Myc-HIPK2 and HA-Ubiquitin (HA-Ub) ±PARP1 WGR, followed by incubation with MG132. Then, immunoprecipitation and immunoblotting were performed as indicated. Asterisk represents ubiquitinated myc-HIPK2. IgG_L_ indicates immunoglobulin light chain

We then questioned whether the activity of PARP1 is required for the degradation of HIPK2. To address this, we used DPQ, a potent and specific inhibitor of PARP1. Interestingly, PARP1-dependent HIPK2 degradation was not inhibited by DPQ (Figure 3a). We also observed that enzymatically inactive PARP1 E988K mutant promoted the degradation of HIPK2 (Figure 3b). These results suggest that PARP1-mediated HIPK2 degradation was independent of PARP1 enzymatic activity.

Thus, we hypothesized that PARP1 may mediate protein interaction required for the HIPK2 degradation. Using a series of C-terminal deletion mutants of PARP1, we found that tryptophan-glycine-arginine (WGR) domain of PARP1 (amino acids 525–692) was sufficient to promote the degradation of HIPK2 (Figure 3c–e). To further examine whether the WGR domain of PARP1 is required for the interaction with HIPK2, we performed co-immunoprecipitation assay using PARP1 WGR domain or WGR deletion mutant (ΔWGR). The WGR domain alone was co-immunoprecipitated with HIPK2 (Figure 3f) but PARP1ΔWGR was not (Figure 3g). Moreover, PARP1ΔWGR failed to reduce the HIPK2 protein levels (Figure 3h). These results indicate that the WGR domain of PARP1 is required for the interaction with HIPK2. In addition, HIPK2 ubiquitination was increased when PARP1 WGR domain was overexpressed (Figure 3i). Taken together, the WGR domain of PARP1 was necessary and sufficient for the promotion of proteasomal degradation of HIPK2.

### HIPK2 interacted with PARP1 via its interaction domain

**Figure 4:**
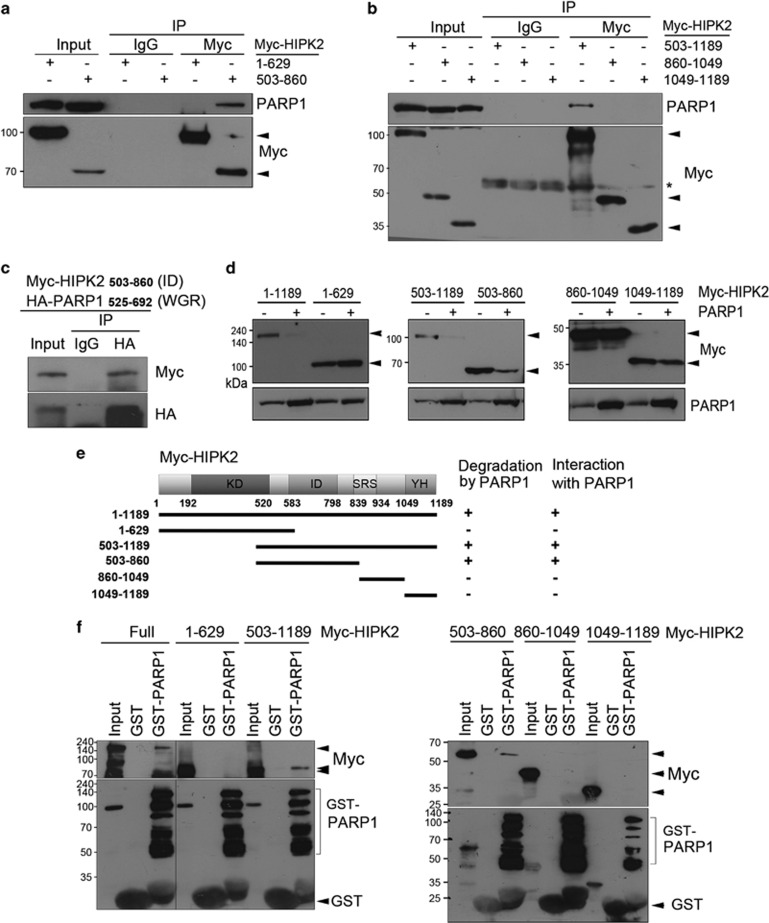
HIPK2 interacted with PARP1 via its ID. (**a** and **b**) HEK 293 cells were transfected with various expression vectors for Myc-HIPK2 (amino acids 1–629, 503–860, 503–1189, 860–1049 and 1049–1189). After immunoprecipitating HIPK2 with anti-Myc antibodies, co-precipitating endogenous PARP1 protein was detected by immunoblotting (upper panels). The blots were also examined for the detection of HIPK2 variants using anti-Myc antibodies (lower panels). (**c**) HEK 293 cells were co-transfected with expression vectors for HA-PARP1 WGR with Myc-HIPK2 503–860. Cell lysates were immunoprecipitated with anti-HA antibodies, followed by immunoblotting with anti-Myc and anti-HA antibodies. (**d**) HEK 293 cells were transfected with expression vectors encoding Myc-HIPK2 full-length (amino acids 1–1189) or deletion mutants (amino acids 1–629, 503–1189, 503–860, 860–1049 and 1049–1189) with or without PARP1 expression vector for immunoblotting. (**e**) A schematic diagram for the deletion mutants of HIPK2 is shown. KD, kinase domain; ID, interaction domain; SRS, speckle-retention signal domain; YH, tyrosine/histidine-rich domain. (**f**) GST and GST–PARP1 proteins were incubated with the indicated *in vitro* translated Myc-HIPK2 deletion mutant proteins. GST pull-downs were analyzed by immunoblotting using anti-Myc antibodies, anti-PARP1 and anti-GST antibodies

We then wanted to identify which domain of HIPK2 interacts with PARP1. To locate the PARP1-interacting domain, full length or truncated HIPK2 (amino acids 1–629, 503–860, 860–1049 and 1049–1189) was examined for the co-immunoprecipitation with PARP1. We found that PARP1 was co-immunoprecipitated with the interaction domain (ID, amino acids 503–860) of HIPK2 (Figure 4a and b). Furthermore, the ID of HIPK2 was co-immunoprecipitated with the PARP1 WGR as well (Figure 4c). We then expressed the deletion mutants of HIPK2 and examined which was decreased by PARP1 (Figure 4d). We observed that PARP1 could not decrease HIPK2 when the ID was absent (Figure 4d and e), confirming the ID is necessary for the PARP1-mediated HIPK2 degradation. We then examined whether HIPK2 ID and PARP1 directly interact by GST pull-down assay. Figure 4f shows that PARP1 directly bound to the ID of HIPK2. These results suggest that HIPK2 and PARP1 directly interact via ID and WGR domain, respectively.

### HSP70 is involved in the PARP1-mediated HIPK2 degradation

**Figure 5:**
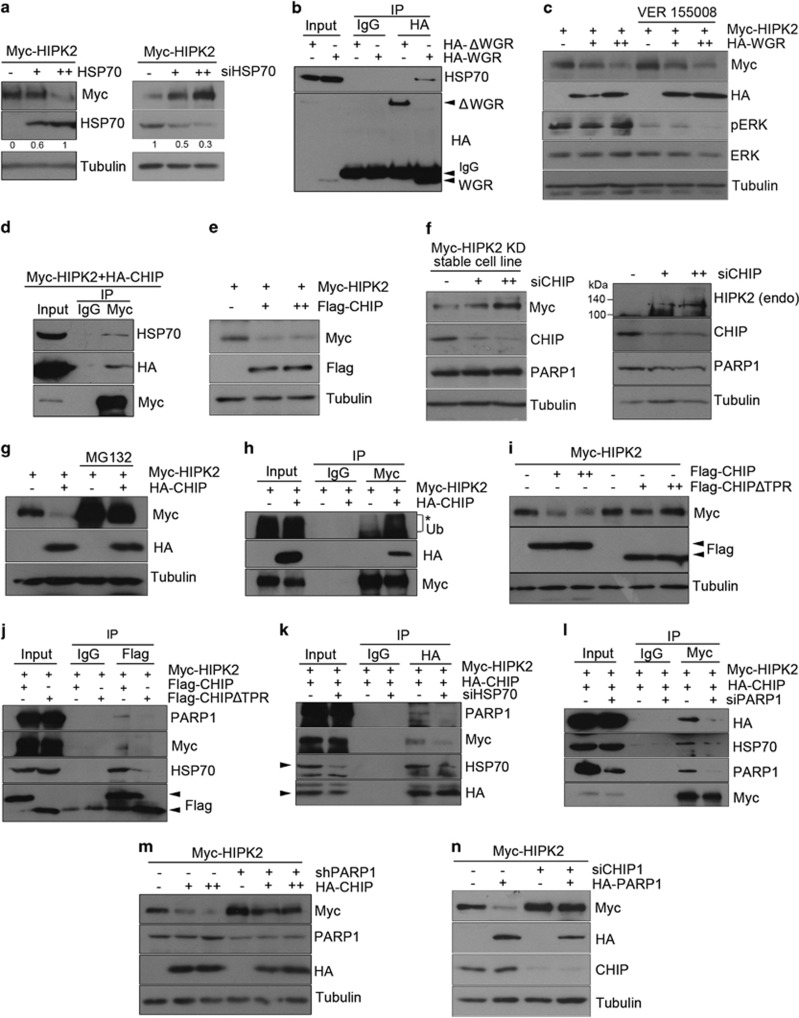
HSP70 and CHIP mediated the HIPK2 degradation promoted by PARP1. (**a**) HEK 293 cells were transfected with expression vectors for Myc-HIPK2 (0.25 *μ*g) and HSP70 (0.25 and 0.5 *μ*g DNA, left panel) or siRNA for HSP70 (50 and 100 pmol, right panel). Then the samples were processed for immunoblotting as indicated. Relative band intensities of HSP70 was determined by densitometry and indicated below. (**b**) HA-PARP1ΔWGR or HA-PARP1 WGR expression vector was transfected into HEK 293 cells for immunoprecipitation/immunoblotting as indicated. (**c**) HEK 293 cells were transfected with expression vectors for Myc-HIPK2 (0.25 *μ*g) and HA-PARP1 WGR (0.25 and 0.5 *μ*g). The cells were treated with 40 *μ*M of VER155008 for 10 h and then processed for immunoblotting. (**d**) Expression vectors for Myc-HIPK2 and HA-CHIP were co-transfected into HEK 293 cells for immunoprecipitation and blotting as indicated. (**e**) HEK 293 cells were transfected with expression vectors for Myc-HIPK2 with Flag-CHIP (0, 0.25 and 0.5 *μ*g) for immunoblotting as indicated. (**f**) HEK 293 cells stably expressing kinase dead HIPK2 (HIPK2 KD, left) or naive HEK 293 cells (right) were transfected with siRNA for CHIP (siCHIP; 50 and 100 pmol) and then processed for immunoblotting. (**g**) HEK 293 cells were transfected with Myc-HIPK2 with or without HA-CHIP expression vectors in the presence or absence of MG132 and then analyzed by immunoblotting. (**h**) HEK 293 cells transfected with Myc-HIPK2 with or without HA-CHIP were immunoprecipitated using anti-Myc antibody and then immunoblotted as indicated. An asterisk indicates the ubiquitinated Myc-HIPK2. (**i**) Myc-HIPK2 expression vector was transfected into HEK 293 cells with increasing amounts of expression vector for either Flag-tagged wild-type CHIP or CHIPΔTPR (TPR-deleted mutants) for the immunoblotting. (**j**) HEK 293 cells were transfected with expression vectors for Flag-CHIP or Flag-CHIPΔTPR in combination with Myc-HIPK2 plasmid and then treated with MG132 (5 *μ*M) for 10 h. Then, the cell lysates were immunoprecipitated and blotted as indicated. (**k** and **l**) HEK 293 cells were transfected with control siRNA, siHSP70 or siPARP1 in combination with Myc-HIPK2 and HA-CHIP expression vectors and then incubated with MG132 (5 *μ*M) for 10 h. Cell lysates were immunoprecipitated and then blotted as indicated. (**m**) Myc-HIPK2 with or without shPARP1 expression vectors were transfected into HEK 293 cells in combination with increasing amounts of HA-CHIP expression vector (0.25 and 0.5 *μ*g). Cell lysates were analyzed by immunoblotting. (**n**) Myc-HIPK2 expression vectors with control siRNA or siRNA for CHIP (siCHIP) were transfected into HEK 293 cells in combination with HA-PARP1 expression vector. Cell lysates were processed for immunoblotting

We observed that WGR domain of PARP1 mediates the promotion of HIPK2 degradation. However, the function of WGR domain in PARP1 is not well identified compared with other domains of PARP1. We postulated that the WGR domain may promote HIPK2 degradation by mediating interaction between HIPK2 and molecules of ubiquitin-proteasome system. Previous studies suggested that HSP70 is involved in the regulation of protein turnover by proteasome.^26^ In addition, HSP70 has been reported to interact with PARP1 and participates in maintaining the genome integrity.^27^ To examine whether HSP70 is involved in the PARP1-mediated HIPK2 degradation, we first tested whether overexpressed HSP70 alters HIPK2 protein levels. Interestingly, the protein level of HIPK2 was reduced by HSP70 overexpression and increased by HSP70 knockdown (Figure 5a). In addition, PARP1 WGR domain interacted with HSP70, whereas PARP1ΔWGR failed to interact with HSP70 (Figure 5b). These results suggest that HSP70 may be involved in the PARP1 WGR-mediated promotion of HIPK2 degradation.

We then investigated whether PARP1 WGR-mediated HIPK2 degradation is regulated by HSP70 chaperone activity using a potent inhibitor of HSP70, VER 155008. The chaperone inhibiting activity of VER 155008 was confirmed by reduction in the ERK phosphorylation.^28^ However, the HSP70 inhibitor did not change the WGR-mediated HIPK2 degradation (Figure 5c). These results suggest that HSP70 is involved in the PARP1-mediated HIPK2 degradation independently of its chaperone activity.

### CHIP mediated the HIPK2 degradation promoted by PARP1

It has been reported that HSP70 binds to the E3 ligase C-terminus of HSP70-interacting protein (CHIP) on tetratricopeptide repeat (TPR) domain^29^ and promotes the ubiquitination of many CHIP substrates.^30, 31^ Therefore, we hypothesized that CHIP may be involved in the HSP70-promoted HIPK2 degradation. To test this possibility, we first examined whether CHIP interacts with HIPK2. Immunoprecipitation assays showed that HIPK2 interacted with CHIP and HSP70 (Figure 5d). Furthermore, overexpression of CHIP markedly decreased the HIPK2 protein amount, whereas knockdown of CHIP increased the protein level of both exogenous kinase-dead HIPK2 in a stable cell line and endogenous HIPK2 (Figure 5e and f). No previous study has suggested CHIP as a ubiquitin ligase for HIPK2. Thus, we then examined whether CHIP decreased the HIPK2 protein amount by promoting ubiquitin-proteasome-mediated degradation. As shown in Figure 5g, CHIP-induced HIPK2 downregulation was effectively abolished by a proteasome inhibitor MG132. In addition, ubiquitination of HIPK2 was promoted by CHIP overexpression (Figure 5h). These results suggest that CHIP can be another ubiquitin ligase for HIPK2.

As mentioned, CHIP binds to HSP70 via its TPR domain.^29^ Therefore, we then wanted to examine whether CHIP-mediated HIPK2 degradation is dependent on the interaction with HSP70. To test this, we examined whether CHIP mutant without TPR domain (ΔTPR) that does interact with HSP70 can decrease the protein level of HIPK2. Unlike wild-type CHIP, ΔTPR failed to decrease the HIPK2 protein amount (Figure 5i), suggesting that CHIP-induced HIPK2 degradation requires CHIP-HSP70 interaction. As CHIP was co-immunoprecipiated with HSP70 and HIPK2 (Figure 5d), we then wanted to examine whether CHIP binds to PARP1 via HSP70. As shown in Figure 5j, CHIP was co-immunoprecipitated with PARP1, as well as HIPK2 and HSP70. However, the CHIPΔTPR did not interact with HIPK2 or PARP1 (Figure 5j and quantification in Supplementary Figure S1), suggesting that CHIP interacts with the PARP1–HIPK2 complex via HSP70. Indeed, HSP70 knockdown resulted in a decrease of CHIP interaction with PARP1 and HIPK2 (Figure 5k and quantification in Supplementary Figure S1). These results suggest that the interaction of CHIP with PARP1 and HIPK2 was mediated by HSP70.

We next examined whether the alteration in the PARP1 expression affects the interaction between HIPK2 and HSP70–CHIP complex. PARP1 knockdown resulted in a marked decrease of HIPK2 interaction with HSP70 and CHIP (Figure 5l and quantification in Supplementary Figure S1). Furthermore, CHIP overexpression failed to decrease HIPK2 protein stability when PARP1 expression was downregulated (Figure 5m). In addition, CHIP knockdown suppressed the PARP1-induced HIPK2 degradation (Figure 5n), suggesting that CHIP and PARP1 are interdependent in promoting HIPK2 degradation.

### PARP1 overexpression suppressed the HIPK2-mediated p53 activation and cell death

**Figure 6:**
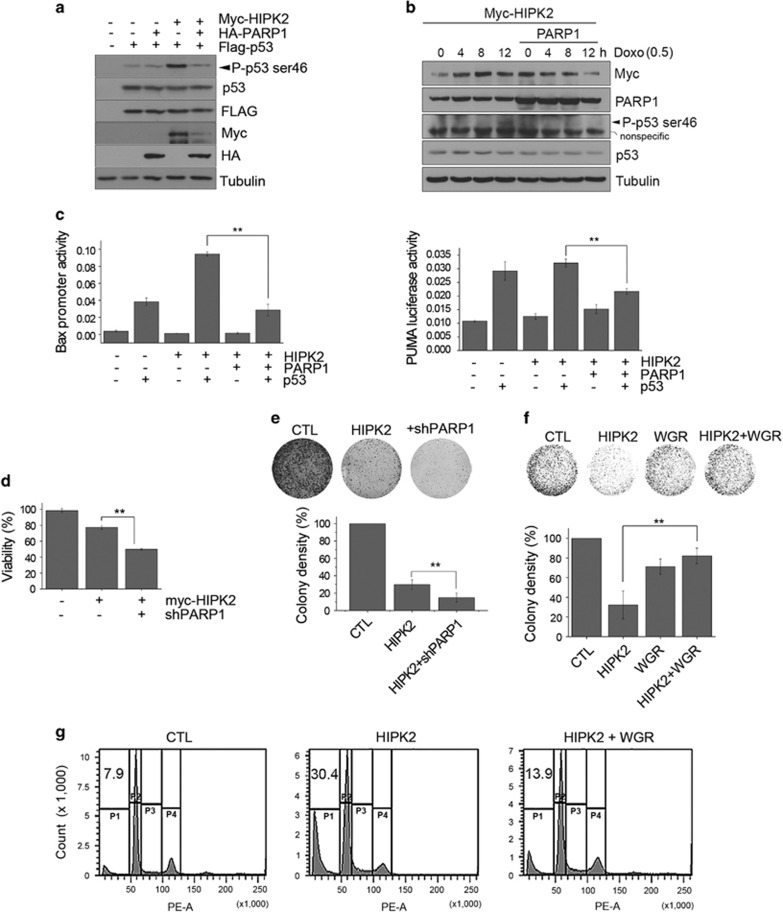
PARP1 overexpression suppressed the HIPK2-mediated p53 activation and cell death. (**a**) H1299 cells were transfected with expression vectors for Myc-HIPK2, HA-PARP1 and Flag-p53 as indicated. Transfected cells were analyzed by immunoblotting as indicated. (**b**) HEK 293 cells were transfected with Myc-HIPK2 with or without PARP1 expression vectors and then treated with a sub-lethal dose of doxorubicin (0.5 *μ*g/ml) for the indicated times. The cells were then analyzed by immunoblotting. (**c**) H1299 cells were transfected with expression vectors for Flag-p53, HA-PARP1 and myc-HIPK2, along with the bax- or puma-luciferase and pRL-TK reporter vector. At 24 h after the transfection, the cells were processed for dual-luciferase assay. Data represent the means±S.D. (*n*=3, **P<0.01). (**d**) HEK 293 cells were transfected with myc-HIPK2 with or without shPARP1 plasmids and then cell viability was measured by MTS assay after 48 h (*n*=3, **P<0.01). (**e**) HCT116 cells were transfected with Flag-HIPK2 expression vector with or without shPARP1. G418 (1 mg/ml)-resistant colonies were stained 4 weeks later. Colony densities were quantified by densitometric reading (bottom panel; *n*=4, **P<0.01). (**f**) HCT116 cells were transfected with the indicated expression vectors and G418-resistant colonies were stained 4 weeks later (upper panels). The colonies were quantified and shown in the lower panel (*n*=3, **P<0.01). (**g**) HCT116 cells were transfected with Flag-HIPK2 with or without WGR expression vector for 48 h and then analyzed by FACS to monitor the cell death. SubG1 fractions are denoted in the upper left corners

In response to severe DNA damage, HIPK2 is stabilized and phosphorylates p53 at serine 46 to activate pro-apoptotic p53-target genes such as *PUMA*, *Bax* and *Noxa*.^9, 10, 32^ Thus, we investigated whether PARP1 can attenuate the p53 phosphorylation induced by HIPK2. Figure 6a shows that overexpression of HIPK2 efficiently promoted the serine 46 phosphorylation of the exogenous p53, whereas co-transfection with PARP1 reduced it in the p53-null H1299 cells. Similar results were obtained when we examined the phosphorylation of endogenous p53 in 293 cells following a sublethal treatment with doxorubicin (Figure 6b). Next, we examined whether PARP1 overexpression can alter the transactivation of p53 proapoptotic target genes by luciferase reporter assay in H1299 cells. As shown in Figure 6c, overexpression of PARP1 suppressed the *Bax* and *PUMA* promoter activation promoted by HIPK2. These results suggest that HIPK2-induced p53 activation was reduced by PARP1 overexpression.

We next investigated whether PARP1 can attenuate the cell death induced by HIPK2. In both cell viability assay (Figure 6d) and colony formation assay (Figure 6e) on HCT116 cells, PARP1 knockdown promoted the decrease of cell viability induced by HIPK2 overexpression. However, cell growth was restored when PARP1 WGR was co-expressed (Figure 6f). In addition, FACS analysis supported that HIPK2-dependent apoptosis was decreased by expression of PARP1 WGR (Figure 6g). Taken together, these results suggest that PARP1 can suppress the cell death induced by HIPK2.

### DNA damage modulated the PARP1-mediated HIPK2 degradation

**Figure 7:**
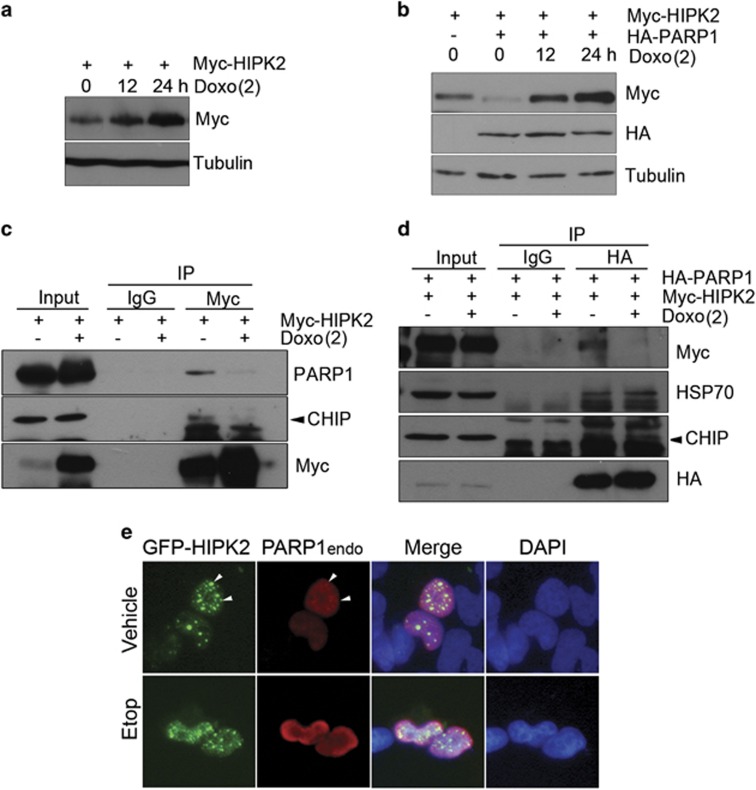
DNA damage modulated the PARP1-mediated HIPK2 degradation. (**a**) HEK 293 cells were transfected with Myc-HIPK2 expression vector and then treated with doxorubicin (Doxo, 2 *μ*g/ml) for 12 and 24 h and then the protein levels of exogenous HIPK2 were examined by immunoblot. (**b**) Myc-HIPK2 expression vector was transfected into HEK 293 cells with or without HA-PARP1 expression vector. At 24 h after the transfection, cells were further incubated with doxorubicin (2 *μ*g/ml) for the indicated times and analyzed by immunoblotting. (**c**) Myc-HIPK2 expression vector was transfected into HEK 293 cells. The transfected cells were treated with MG132 (5 *μ*M) plus or minus doxorubicin (2 *μ*g/ml) for 12 h. Then the cell lysates were immunoprecipitated and then blotted as indicated. (**d**) To monitor the changes in the interaction between HIPK2 and PARP1 after DNA damage, HEK 293 cells were transfected with HA-PARP1 and Myc-HIPK2 and then incubated with vehicle or doxorubicin (Doxo) (2 *μ*g/ml) in the presence of MG132 for 6 h. The cells were processed for immunoprecipitation and immunoblotting. (**e**) To examine the changes in the subcellular localization, HEK 293 cells were transfected with GFP-HIPK2 and then treated with etoposide (Etop, 40 *μ*M) for 24 h. Then, the cells were immunostained using anti-PARP1 antibody

We then examined whether DNA damage can suppress the PARP1-mediated HIPK2 degradation. We confirmed that HIPK2 protein level increased after doxorubicin treatment (Figure 7a). Importantly, a lethal dose of doxorubicin treatment resulted in the accumulation of HIPK2 protein even when PARP1 was overexpressed at 24 h after the treatment (Figure 7b), suggesting that HIPK2 can escape from the PARP1-mediated degradation under DNA damage condition. The doxorubicin treatment induced the cleavage of PARP1 at 24 h but the decrease in the amount of full-length PARP1 became evident at 48 h after the treatment (Supplementary Figure S2).

Furthermore, interaction between HIPK2 and PARP1/CHIP was dramatically decreased as assessed by Myc-HIPK2 immunoprecipitation after doxorubicin treatment, indicating that PARP1 was dissociated from HIPK2 after DNA damage (Figure 7c and quantification in Supplementary Figure S1). When we examined the changes in the interaction by PARP1 immunoprecipitation, the portion of PARP1-bound HIPK2 was again reduced after the doxorubicin treatment (Figure 7d and quantification in Supplementary Figure S1). However, the interaction between PARP1 and CHIP or HSP70 did not change notably after the drug treatment (Figure 7d). When we examined the localization of HIPK2 and PARP1 by immunostaining, etoposide treatment resulted in the change of PARP1 subcellular localization. PARP1 was found concentrated in the nuclear margin following etoposide treatment and few cells exhibited colocalization of HIPK2 and PARP1 (Figure 7e). Taken together, these results imply that the interaction between HIPK2 and PARP1 can be disrupted when the cellular condition requires the accumulation of HIPK2 such as in DNA damage condition.

## Discussion

HIPK2 is a key regulator of DNA damage-induced apoptosis. Following DNA damage, activated HIPK2 inhibits the progression of cell cycle or induces apoptosis via phosphorylation of target proteins such as p53 and CtBP.^9, 10, 33^ Therefore, the expression level and activity of HIPK2 should be regulated for the determination of cell fate between survival and death. Previous studies established that HIPK2 protein stability is tightly regulated by ubiquitin-proteasome system.^3, 4, 5^ In the present study, we have shown that PARP1 promotes the degradation of HIPK2 by recruiting ubiquitin ligase CHIP. By regulating HIPK2 protein stability, PARP1 may have a role in cellular decision-making between DNA repair and apoptosis.

PARP1 is a multifunctional nuclear enzyme that keeps cellular homeostasis by regulating DNA repair, transcription and chromatin modulation by attaching poly(ADP-ribose) (PAR) on target proteins.^11^ Although not much has been known regarding the involvement of PARP1 in the regulation of protein turnover, there are PAR-dependent E3 ubiquitin ligases such as ring finger protein 146 and ubiquitin-like with PHD and RING finger domains 1.^34, 35^ These PAR-binding E3 ligases regulate degradation of PARP1 itself or PARylated proteins and control DNA damage response or heterochromatin silencing.^34, 35^ In our study, PARP1 enzymatic activity was not required for the promotion of HIPK2 degradation. Instead, WGR domain interacted with HSP70/CHIP to promote the proteasomal degradation of HIPK2. The WGR domain is the least characterized among the domains of PARP1. A couple of studies have suggested that WGR domain is necessary for the PARP1 enzymatic activity.^36, 37, 38^ However, our data showed that WGR domain has discrete function apart from catalytic activation of PARP1. We found that HSP70 specifically interacted with the WGR domain and recruited CHIP. Previous reports have suggested that PARP1 does have an activity-independent and interaction-mediated function.^39^ Our present work defines a novel function of PARP1 and WGR domain mediated by protein–protein interaction.

PARP1 has long been known as a genomic guardian.^13^ PARP1 knockout cells exhibit higher rate of sister chromatid exchange and enhanced sensitivity towards DNA-damaging agents.^40^ In addition, PARP1 has been shown to act as a molecular platform for recruiting many proteins involved in DNA repair or metabolism.^13^ Therefore, basal level of PARP1 activity is required for the survival and homeostasis of cells. Our results indicating PARP1 protein promotes the proteasomal degradation of an apoptosis inducer HIPK2 further support the notion that PARP1 has prosurvival function in unstressed conditions. Degradation of another tumor suppressor phosphatase and tensin homolog (PTEN) has been shown to be promoted by tankyrase, a PARP family enzyme.^41^ It would be interesting to test the possibility that PARP1 promotes the degradation of other tumor suppressors in unstressed conditions.

It has been reported that HIPK2 has several ubiquitin ligases acting in different cellular conditions. In unstressed conditions, SIAH1, WSB1 and SCF/Fbx3 promote the steady-state degradation of HIPK2 to prevent cell cycle arrest or apoptosis.^4, 5^ Under hypoxic conditions, SIAH2 induces proteasomal degradation of HIPK2.^3^ In the present study, we identified CHIP as an E3 ubiquitin ligase for HIPK2. CHIP has been suggested to set the balance between protein folding by chaperone system and proteasomal degradation of misfolded proteins.^42^ However, CHIP promotes the degradation of not just misfolded proteins but also many signaling molecules that need tight regulation of their protein stability. For example, CHIP mediates the proteasomal degradation of tumor suppressor PTEN, p53, inducible nitric oxide synthase and apoptosis signal regulating kinase 1 to least a few.^42^ By regulating the stability of both oncogenic and tumor suppressor proteins, CHIP has been implicated in tumorigenesis.^43^ Our results suggest that CHIP regulates the degradation of HIPK2 by recruitment to PARP1 via HSP70. Thus, it can be postulated that CHIP may function as a prosurvival factor in regard to HIPK2 regulation.

Previous studies have shown that HIPK2 phosphorylates the tumor suppressor p53 at serine 46, which in turn initiates apoptosis following lethal DNA damage.^9, 10, 44^ In accordance with this, we observed the phosphorylation of p53 at serine 46 and induction of cell death when HIPK2 was overexpressed. However, this was all suppressed by PARP1 co-expression. However, HIPK2 needs to be accumulated to induce cell cycle arrest or cell death under lethal DNA damage condition. Indeed, our data suggest that HIPK2 escapes from the PARP1-mediated degradation under DNA damage condition. Following doxorubicin treatment, the protein level of HIPK2 still increased even when PARP1 was overexpressed and the interaction between HIPK2 and PARP1 was abolished (Figure 7). We postulate that DNA damage-induced modification on either PARP1 or HIPK2 may weaken their association. PARP1 PARylates on itself when activated by DNA damage and PARylation often disrupts protein–protein interaction.^11, 12, 13^ Another possibility is that phosphorylation by DNA damage sensor kinases such as ATM or ATR weakens the interaction between HIPK2 and PARP1. The interaction between HIPK2 and SIAH1 has been shown to be disrupted by ATM/ATR.^4, 5^ Furthermore, it has been reported that PARP1 interacts with ATM although phosphorylation of PARP1 by ATM has not been shown.^45^ Thus, it is possible that activation of ATM results in the dissociation of PARP1 and HIPK2. It remains to be studied how the interaction between PARP1 and HIPK2 is disrupted.

Taken together, our results suggest that PARP1 may play a critical role in setting the gear toward initiation of apoptosis *versus* damage repair by promoting HIPK2 degradation. As HIPK2 is a tumor suppressor and mediator of DNA damage-induced apoptosis, our results propose a new measure to improve the efficiency of genotoxic cancer therapies possibly by interfering with the PARP1 WGR-HIPK2 interaction.

## Materials and Methods

### Reagents

Cell lines were purchased from ATCC (Manassas, VA, USA). All the culture medium and supplements were purchased from JBI (Daegu, Korea). All other reagents were purchased from Sigma-Aldrich (St. Louis, MO, USA), unless stated otherwise.

### Antibodies

The following antibodies were used: mouse monoclonal anti-PARP1 antibody (BD Bioscience, San Jose, CA, USA), mouse monoclonal anti-HIPK2 antibody^46^ (kindly provided by Dr H Koseki, RIKEN Research Center, Yokohama, Japan), rabbit polyclonal anti-PAR (BD Bioscience), mouse monoclonal anti-*α*-tubulin (Sigma-Aldrich), mouse monoclonal antibodies for Myc, HA, Flag and GST (abm, Richmond, Canada), rabbit polyclonal anti-phospho-p53 (serine 46) (BD Bioscience), mouse monoclonal anti-ubiquitin (Millipore, Billerica, MA, USA), rabbit monoclonal anti-p53 (Cell Signaling, Danvers, MA, USA), rabbit polyclonal anti-HSP70 (Stressgen, San Diego, CA, USA), rabbit polyclonal anti-CHIP (kindly provided by Dr S Yoo, Kyung Hee University, Korea). Specificity of the antibody that reacts with the endogenous HIPK2 protein was confirmed by detecting the accumulated endogenous HIPK2 following DNA damage and the exogenously expressed HIPK2 (Supplementary Figure S3).

### Cell culture and transfection

HEK 293, HCT116 and H1299 cells were maintained in recommended medium supplemented with 10% fetal bovine serum and 1% antimycotics solution at 37 °C in a 5% CO_2_ incubator. Viability of the cells was measured using CellTiter 96 AQueous One Solution assay kit (Promega, Madison, WI, USA).

For the transient expression of each expression vector (1 *μ*g of DNA/35 mm dish), transfection was performed using 25 kDa linear form polyethylenimine (3 *μ*g/1 *μ*g of DNA) according to the manufacturer's protocol (Polysciences, Niles, IL, USA).

### Plasmid construction and site-directed mutagenesis

The full-length human Myc-HIPK2, GFP-HIPK2 and various HIPK2 deletion constructs were described previously.^47^ The PARP1 and CHIP expression plasmids were purchased from Korea Human Gene Bank (Daejeon, Korea). Various PARP1 deletion mutants were constructed by insertion of each PCR-amplified DNA fragments into the HindIII and XhoI sites of HA-pcDNA3.0. The GST-PARP1 was generated by cloning PARP1 cDNA into the pGEX-4T-1 vector. The active site mutant of PAPR1 (E988K) was generated using the QuickChange mutagenesis kit (Stratagene, La Jolla, CA, USA) according to the manufacturer's protocol.

### RNA interference

The pLKO.1 lentiviral vectors containing siRNA of PARP1 targeting sequence were purchased from Open Biosystems (Pittsburgh, PA, USA). The PARP1 siRNA (5′-CUCUCAAAUCGCUUUUACA-3′), CHIP siRNA (5′-CGC UGG UGG CCGUGU AUU A-3′) and negative control siRNA were purchased from Bioneer (Daejeon, Korea). The specificity of the siCHIP has been confirmed and described by Lee *et al.*^48^ For HSP70 knockdown, the siRNA (5′-CCUGAUGGUAAUUAGCUGG-3′) was purchased from Ambion (Austin, TX, USA). siRNA (50–100 pmol) was transfected using the Lipofectamine 2000 (Invitrogen, Carlsbad, CA, USA) according to the manufacturer's protocol.

### Generation of stable cell line

Expression vector carrying Myc-HIPK2 kinase-dead mutant (K221R) was generated by cloning PCR-generated 6 × Myc tag fragments into HIPK2 K221R expression plasmid described previously.^47^ The Myc-HIPK2 K221R expression vector was transfected into 293 cells. The transfected cells were selected with G418 (0.2~1 mg/ml) and cultured for 4 weeks. Batch cultured cells were used for experiments.

### Immunocytochemistry

For the immunostaining, HEK 293 cells were grown on poly-L-lysine-coated 12 mm coverslips in 24-well plates and transfected with 0.25–0.5 *μ*g of expression plasmids. Twenty-four hours after the transfection, the cells were fixed with 4% paraformaldehyde in PBS for 10 min at room temperature and permeabilized with PBS containing 0.5% Triton X-100 for 20 min. After washing with PBS three times, cells were blocked for 1 h with normal goat serum (NGS, 10%) in PBS containing 0.1% Triton X-100 (PBST). Next, the cells were incubated with primary antibodies diluted in 0.1% PBST containing 5% NGS at 4 °C overnight. The samples were washed for 5 min in 0.1% PBST three times. The cells were then incubated with biotin-conjugated secondary antibodies. After washing, biotin-labeled samples were incubated with FITC- or Texas Red-conjugated streptavidin (Molecular Probes, Eugene, OR, USA) for 30 min at room temperature. After final washing, samples were mounted with mounting medium with DAPI (Slowfade Gold reagent with DAPI, Invitrogen). The samples were examined under a fluorescence microscope (Axioplan 2, Zeiss, Jena, Germany).

### Immunoprecipitation and immunoblotting

The co-immunoprecipitation was performed using 2 × 10^7^ cells in high-salt lysis buffer (50 mM Tris/HCl pH 7.5, 10% glycerol, 1% Nonidet P-40, 300 mM NaCl, 150 mM KCl, 5 mM EDTA, 1 mM dithiothreitol, 10 mM NaF, 0.5 mM sodium vanadate, 10 *μ*g/ml leupeptin, 10 *μ*g/ml aprotinin and 1 mM PMSF). After incubation on ice for 10 min and centrifugation at 10 000 × *g* for 10 min at 4 °C, equal volumes of protein were diluted with lysis buffer without NaCl and KCl, then incubated overnight with antibodies and protein A-Sepharose beads at 4 °C on a rotating wheel. The beads were washed three times with lysis buffer. The whole-cell lysates or immunoprecipitates were boiled for 5 min at 95 °C in 2 × SDS sample buffer (0.25 M Tris-HCl pH 6.8, 2% SDS, 10% 2-mercaptoethanol, 30% glycerol and 0.01% bromophenol). Immunoblotting was carried out by conventional methods. Immunoblottings for tubulin or actin served as loading controls throughout the study.

### Reverse-transcription PCR

Total RNA was isolated using RNeasy minikit (Qiagen, Valenia, CA, USA). cDNA was synthesized using Moloney Murine Leukemia Virus Reverse Transcriptase (Promega) according to the manufacturer's protocol. The cDNA products from the reverse transcription reaction were used as templates for PCR using the following primer pairs: mouse HIPK2 (forward 5'-CTT CAG GAG CCA TCG CCT AC-3′, reverse 5′-CTG TTG TGC GGGAAG GTG TA-3′), human HIPK2 (forward 5'-AGT CCA CGA CTC CCC CTA CT-3', reverse 5'-ATG GTG GGA GTG ATG TAG GC-3'), PARP1 (forward 5'-TTGCAA GAA ATG CAG CGA GAG-3', reverse 5'-GAT GGT ACC AGC GGT CAA TCA-3') and β-actin (forward 5′-CCT CGC CTT TGC CGA TCC-3′, reverse 5′-GGA TCT TCA TGA GGT AGT CAG TC-3′). PCR reactions were performed using the following conditions: 1 min 95 °C, 1 min 57 °C, 1 min 72 °C, 24 cycles. PCR products were analyzed on 2% agarose gels.

### *In vitro* GST pull-down assay

The GST-fusion proteins were expressed in BL21 *Escherichia coli* and were purified with glutathione-Sepharose beads (Peptron, Daejeon, Korea) according to the protocol provided by the manufacturer. The various proteins were *in vitro* translated by using the coupled TnT *in vitro* transcription–translation system from rabbit reticulocyte lysates in accordance with the instructions of the manufacturer (Promega). *In vitro* translated proteins were incubated with GST-fusion proteins at 4 °C for 2 h in binding buffer (20 mM Tris/HCl pH 8.0, 10% (v/v) glycerol, 0.2% (v/v) NP-40, 0.5 mM EDTA, 1 mM dithiothreitol and 200 mM NaCl) and washed three times in PBS with 0.5% (v/v) Triton X-100. After washing, the bound proteins were eluted with 2 × SDS sample buffer, separated by denaturing SDS-PAGE and analyzed by immunoblotting.

### Luciferase assay

To measure Bax or PUMA promoter activity, H1299 cells were transfected with expression vectors encoding p53, PARP1 and HIPK2, along with the PUMA- or Bax-luciferase reporter vector (kindly provided by Dr G D'Orazi, Regina Elena Cancer Institute, Rome, Italy) and control pRL-TK plasmids. At 24 h after the transfection, dual luciferase activity assay was carried out using dual luciferase reporter assay kit (Promega) according to the manufacturer's protocol.

### Colony formation assay

HIPK2 expression vector (pCMV-Flag) was transfected into HCT116 cells, with or without PARP1 expression vector for 24 h. Next, the cells were selected by G418 (1 mg/ml) for 4 weeks. Surviving colonies were stained with crystal violet.

### Cell cycle analysis

HCT116 cells were transfected with Flag-HIPK2 with or without PAPR1 WGR expression vector. After 48 h, the transfected cells were fixed in 70% ethanol at 4 °C overnight and then stained with 10 *μ*g/ml propidium iodide in the presence of 50 *μ*g/ml RNase A. The cells were analyzed by flow cytometry (FACSCanto 2, BD).

### Statistics

For the statistical analysis, all the experiments were repeated at least three times. The results were expressed as mean±S.D. of at least three independent experiments, unless stated otherwise. Paired data were evaluated by Student's *t*-test. A value of *P*<0.05 was considered significant.

## Abbreviations

HIPK2: homeodomain-interacting protein kinase 2
PARP1: poly(ADP-ribose) polymerase 1
CHIP: C-terminus of HSP70-interacting protein
SIAH1: seven in absentia homolog 1
WSB1: WD40 repeat/SOCS box-containing protein 1
ATM: ataxia telangiectasia mutated
ATR: ATM and Rad3-related
CtBP: C-terminal binding protein
HIF1: hypoxia-inducible factor 1
TPR: tetratricopeptide repeat
PTEN: phosphatase and tensin homolog
PAR: poly(ADP-ribose)
