# Supplementary material for: PARP1 regulates the protein stability and proapoptotic function of HIPK2
Source: Cell Death Dis. 2016 Oct 27;7(10):e2438–. doi: 10.1038/cddis.2016.345 (PMC5134000; doi:10.1038/cddis.2016.345)
Supplement: Supplementary Figure Legends [file cddis2016345x1.docx]

**Supplementary figure legends**

**Supplementary Figure S1** Quantification of the coimmunoprecipitated proteins. Relative amounts of the coimmunoprecipitated proteins in Figures 5j (a), 5k (b), 5l (c), 6c (d), and 6d (e) were quantified by densitometric readings of the immunoblot bands (n=3, ** p<0.01, *** p<0.001).

**Supplementary Figure S2** Cleavage of PARP1 following DNA damage. 293 cells were incubated with doxorubicin (2 μg/ml) for the indicated times and then processed for the immunoblotting analysis using antibodies as indicated.

**Supplementary Figure S3** Validation of the anti-HIPK2 antibody that detects endogenous form. (a) 293 cells were treated with a DNA damaging reagent doxorubicin for the indicated times and then processed for the immunoblot using anti-HIPK2 antibody. Note the antibody detected HIPK2 at around 130 kDa after 24 h doxorubicin treatment. (b) 293 cells were transfected with Myc-HIPK2 expression vector and then immunoblotted using HIPK2 antibody (top panel, asterisk indicates overexposure to visualize low level of endogenous HIPK2 in the control vector-transfected cells). The membrane was then stripped and reprobed using anti-Myc antibody. Blots for tubulin served as loading controls.
