# Supplementary figures and images for: PARP1 regulates the protein stability and proapoptotic function of HIPK2
Source: Cell Death Dis. 2016 Oct 27;7(10):e2438–. doi: 10.1038/cddis.2016.345 (PMC5134000; doi:10.1038/cddis.2016.345)

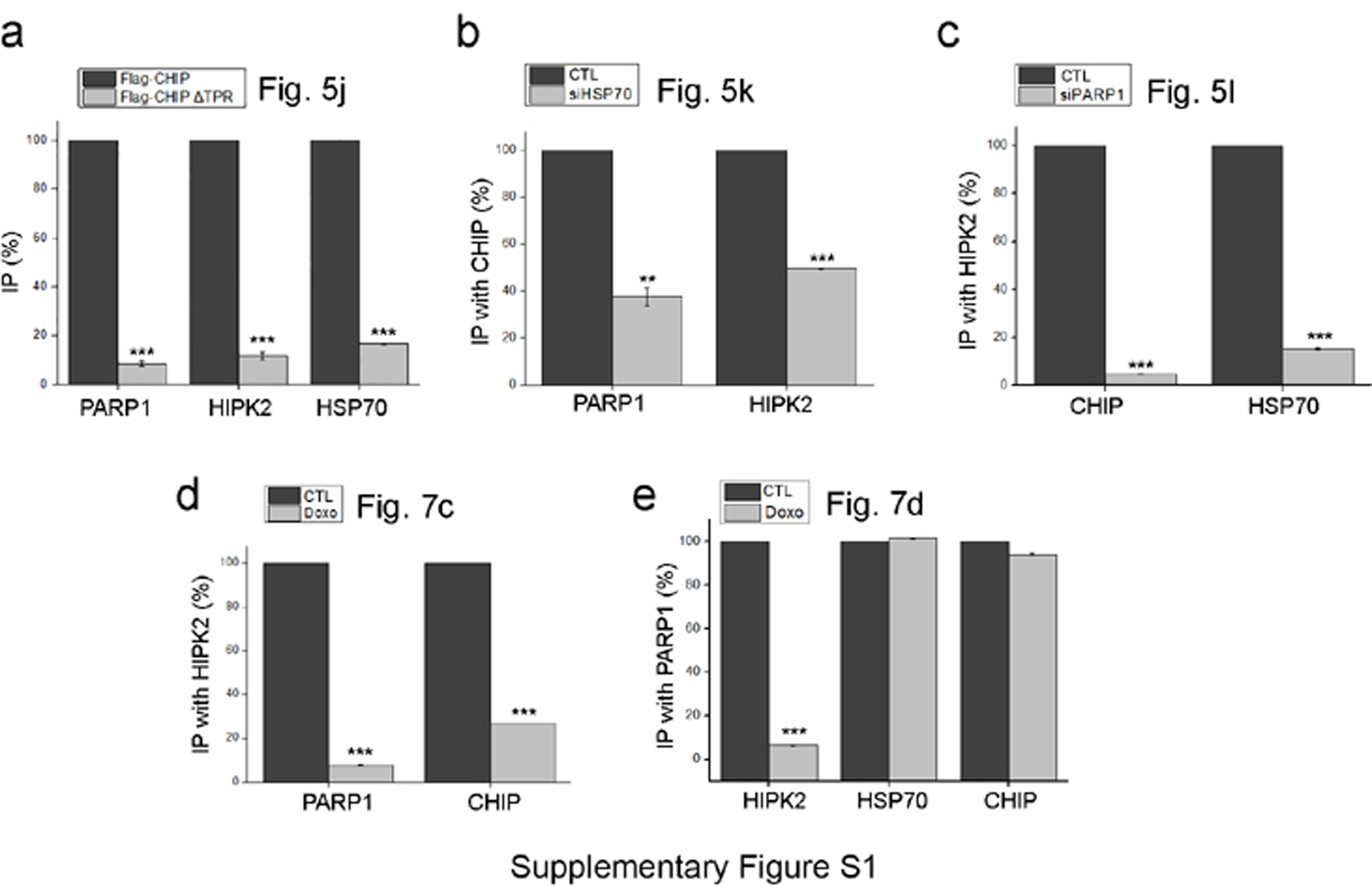

Supplement: Supplementary Figure S1 [file cddis2016345x2.tif]

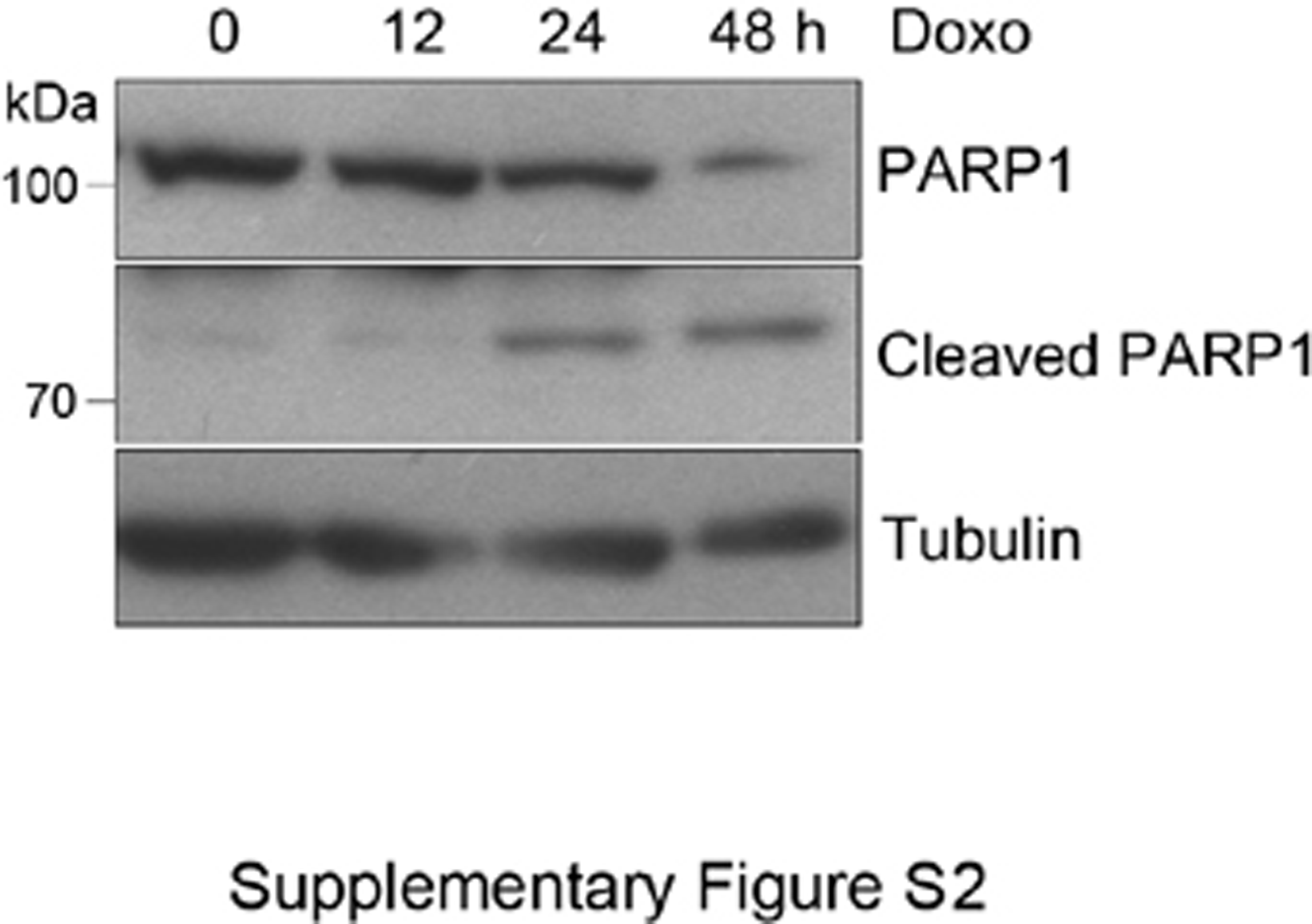

Supplement: Supplementary Figure S2 [file cddis2016345x3.tif]

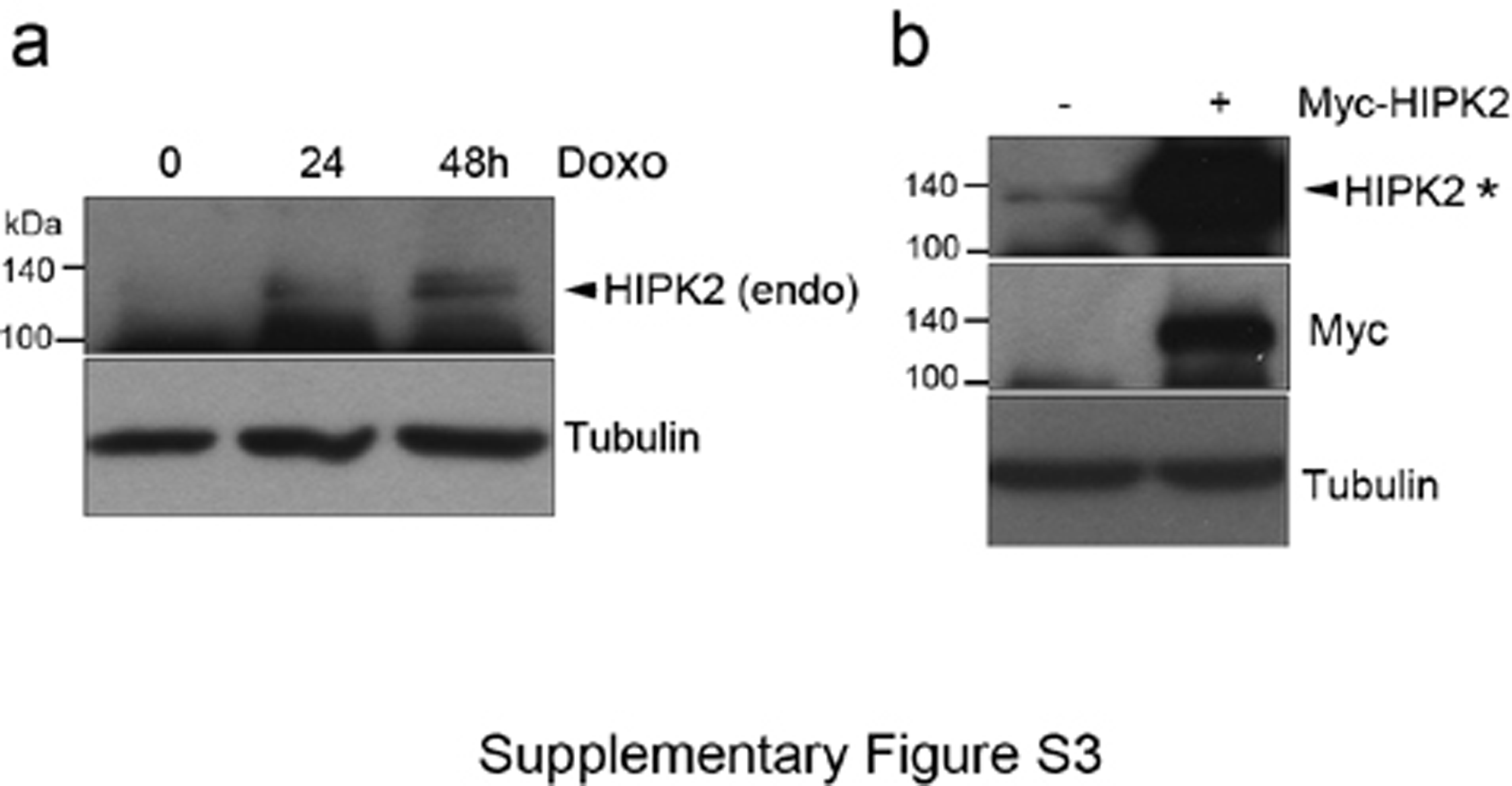

Supplement: Supplementary Figure S3 [file cddis2016345x4.tif]
